# Diagnostic utility of contrast-enhanced ultrasound parameters in classifying lupus nephritis

**DOI:** 10.1093/ckj/sfaf314

**Published:** 2025-10-08

**Authors:** Shuping Wei, Yidan Zhang, Chunrui Liu, Baojie Wen, Jing Yao, Zhichao Xia, Xue Xu, Zhibin Jin

**Affiliations:** Department of Ultrasound, Nanjing Drum Tower Hospital, Affiliated Hospital of Medical School, Nanjing University, Nanjing, Jiangsu, China; Department of Ultrasound, Nanjing Drum Tower Hospital, Affiliated Hospital of Medical School, Nanjing University, Nanjing, Jiangsu, China; Department of Ultrasound, Nanjing Drum Tower Hospital, Affiliated Hospital of Medical School, Nanjing University, Nanjing, Jiangsu, China; Department of Ultrasound, Nanjing Drum Tower Hospital, Affiliated Hospital of Medical School, Nanjing University, Nanjing, Jiangsu, China; Department of Ultrasound, Nanjing Drum Tower Hospital, Affiliated Hospital of Medical School, Nanjing University, Nanjing, Jiangsu, China; Department of Domestic Clinical Application, Shenzhen Mindray Bio-Medical Electronics Co. Ltd, Shenzhen, Guangdong, China; Department of Rheumatology and Immunology, Nanjing Drum Tower Hospital, Affiliated Hospital of Medical School, Nanjing University, Nanjing, Jiangsu, China; Department of Ultrasound, Nanjing Drum Tower Hospital, Affiliated Hospital of Medical School, Nanjing University, Nanjing, Jiangsu, China

**Keywords:** contrast-enhanced ultrasound, lupus nephritis, proliferative, renal biopsy, time-intensity curve

## Abstract

**Background:**

Lupus nephritis (LN) is a severe complication of systemic lupus erythematosus (SLE), with renal biopsy as the diagnostic gold standard. However, biopsy is invasive. This study aims to evaluate the potential of contrast-enhanced ultrasound (CEUS) quantitative parameters as non-invasive predictors in differentiation proliferative LN from non-proliferative LN.

**Methods:**

Fifty-eight biopsy-confirmed LN patients who underwent CEUS within 3 days before biopsy were included retrospectively. Patients were categorized into 38 cases of proliferative LN (Class III, IV, and III/IV + V) and 20 cases of non-proliferative LN (Class II and purely Class V). The clinical and laboratory data, conventional US parameters, and CEUS quantitative parameters derived from time-intensity curves (TICs) were analyzed. Multivariate logistic regression and receiver operating characteristic (ROC) curve analysis were performed to determine significant predictors and evaluate the diagnostic performance.

**Results:**

Patients with proliferative LN exhibited significantly higher absolute time to peak (∆TTP), half descending time (DT/2) and TIC area under curve (TIC-AUC) values than non-proliferative LN patients (*P *< .05). Logistic regression analysis identified TIC-AUC and anti-dsDNA antibody as independent predictors of proliferative LN. ROC analysis revealed that anti-dsDNA positive had an AUC of 0.745, with sensitivity of 87.5% and specificity of 61.5% for predicting proliferative LN. For TIC-AUC, a cutoff value of 8049.0 yielded an AUC of 0.810, sensitivity of 68.8% and specificity of 84.6% for predicting proliferative LN.

**Conclusions:**

CEUS quantitative parameters, particularly TIC-AUC, provide a non-invasive approach for identifying proliferative LN, and complement conventional laboratory markers. These findings demonstrate the potential of CEUS in improving LN diagnosis and facilitating clinical evaluation.

## GRAPHICAL ABSTRACT

**GRAPHICAL ABSTRACT:**
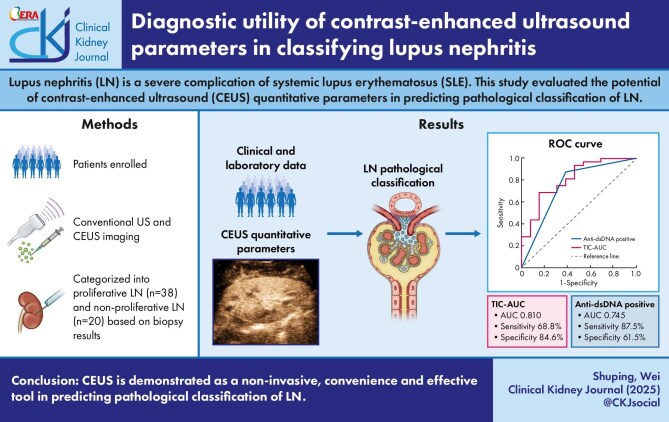

KEY LEARNING POINTS **What was known:**

- Lupus nephritis (LN) is a common and serious complication of systemic lupus erythematosus.
- Renal biopsy is the gold standard for diagnosis, but it is invasive.
- Contrast-enhanced ultrasound (CEUS) has been proven effective in assessing microcirculation in kidney diseases, but its application in LN remains underexplored.

**This study adds:**

- This study demonstrated the diagnostic value of CEUS quantitative parameters in distinguishing between proliferative and non-proliferative LN, providing a potential non-invasive diagnostic tool for LN.

**Potential impact:**

- CEUS could serve as a valuable non-invasive tool for assessing LN, reducing the reliance on invasive biopsies and its associated risks, particularly in patients with contraindications to biopsy.
- CEUS may facilitate earlier detection and better monitoring of LN, ultimately leading to improved patient management and outcomes.

## INTRODUCTION

Systemic lupus erythematosus (SLE) is an autoimmune disease that impacts multiple organs and tissues. Up to 50%–70% of SLE patients experience various degrees of kidney damage. Lupus nephritis (LN) is the major contributor of morbidity and mortality in SLE patients [1]. Accurate diagnosis of renal involvement and close monitoring of disease progression are essential for effective management and treatment of LN [2, 3].

Renal biopsy is considered as the gold standard for diagnosing LN. According to the classification of the International Society of Nephrology and Renal Pathology Society (ISN/RPS), pathological typing of LN, along with assessing the degree of renal inflammation, damage, as well as the activity and chronicity of the disease, can help guide treatment and predict prognosis [4, 5]. Among these types, Class III, Class IV and Class III/IV + V, which are known as proliferative LN, tending to have a more aggressive course and poorer prognosis, requiring more intensive immunosuppressive therapy to prevent irreversible kidney damage, compared with non-proliferative LN, which includes Class I, Class II and purely Class V [6, 7]. Therefore, accurately identifying the pathological type of LN is crucial for guiding clinical treatment and prognosis assessment.

Although renal biopsy plays a vital role in the diagnosis and management of LN, it presents certain challenges. First, renal biopsy is an invasive procedure with potential risks of complications, especially in SLE patients who often have coagulation disorders, increasing the risk of bleeding complications from the procedure, particularly for less experienced physicians [8, 9]. Additionally, repeat biopsies may be required during follow-up if the treatment is not effective or if there is a suspicion of disease transformation [10, 11]. Furthermore, renal biopsy has inherent sampling limitations and may not fully reflect renal tissue changes. This is especially problematic in advanced stages of LN, when significant renal fibrosis may prevent the acquisition of adequate glomerular samples, potentially leading to diagnostic inaccuracies. Consequently, the development of a novel, effective and non-invasive method for early differentiation proliferative from non-proliferative LN, as well as for prognostic assessment, holds significant clinical importance.

Traditional biomarkers for LN, such as anti-dsDNA/C1q antibodies, complement components, and 24-h proteinuria, have limited ability to accurately predict the type of LN. Furthermore, challenges remain in identifying novel biomarkers for diagnosing renal damage and evaluating prognosis in LN [12, 13]. Among imaging modalities, ultrasound (US) plays a vital role in the evaluation of kidney diseases. Compared with computed tomography and magnetic resonance imaging, US offers distinct advantages, including convenience, cost-effectiveness, non-invasiveness, absence of radiation and repeatability, making it the preferred imaging method for LN patients.

The occurrence and progression of chronic kidney disease are closely related to alterations in renal blood perfusion. In recent years, contrast-enhanced ultrasound (CEUS) has been widely used in the evaluation of microcirculation and blood perfusion in various organs, including the renal cortical perfusion [14]. Since the US contrast agent is excreted through the lungs, it is non-nephrotoxic, making it safe for patients with kidney disease. Many studies have demonstrated its effectiveness in diagnosing and evaluating diffuse kidney diseases, such as acute kidney injury, chronic kidney disease and diabetic nephropathy, and in evaluating the functionality of transplanted kidneys [15–17]. However, there have been no studies specifically focusing on the use of CEUS to evaluate renal blood perfusion in LN. Understanding the correlations between CEUS quantitative parameters and the histopathological features of LN can aid the development of methods to guide SLE treatment in clinical practice. Therefore, the purpose of this study is to preliminarily explore the potential of CEUS quantitative parameters in predicting pathological classification of LN.

## MATERIALS AND METHODS

### Patients

This study protocol conformed to the ethical guidelines of the Declaration of Helsinki and was approved by the Ethical Committee of the Nanjing Drum Tower Hospital, Affiliated Hospital of Medical School, Nanjing University (No. 2022–259). Written informed consent was not required due to the retrospective nature of this study.

**Figure 1:**
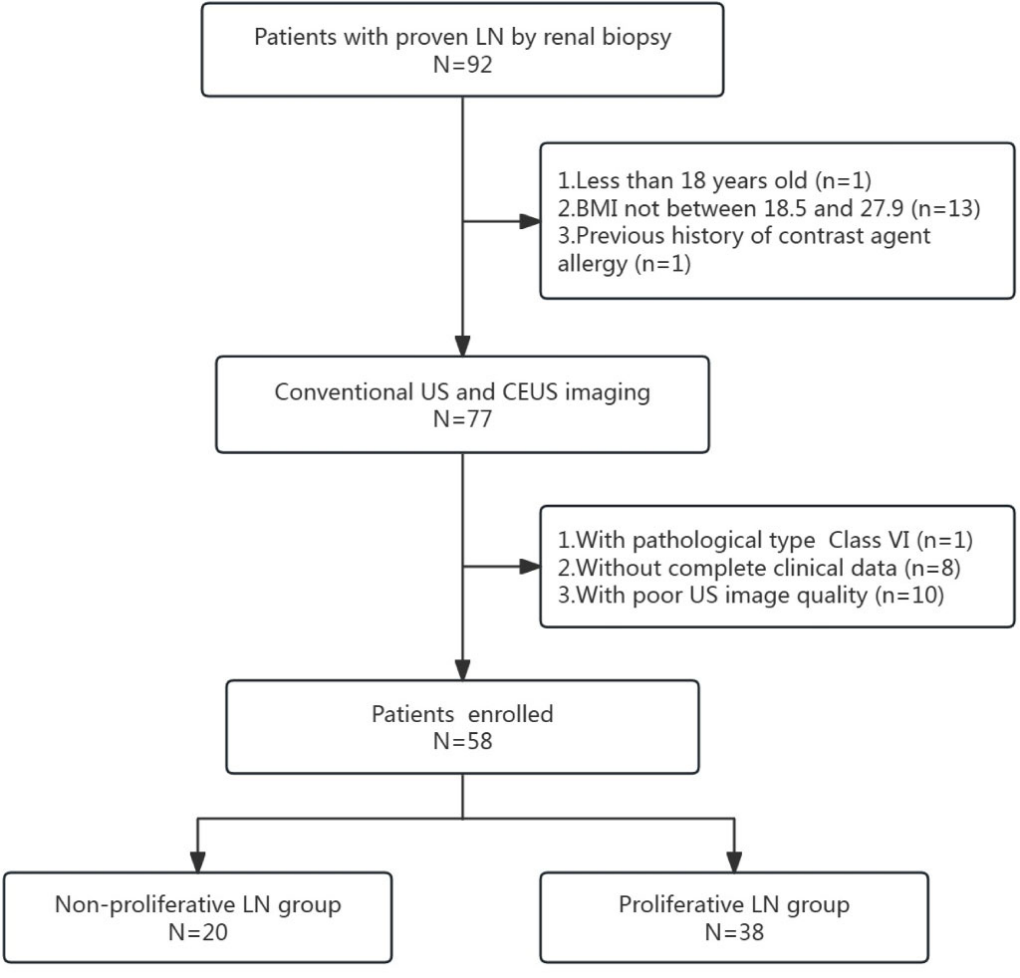
The flowchart of the study.

We enrolled LN patients who underwent renal biopsy in Nanjing Drum Tower Hospital, Affiliated Hospital of Medical School, Nanjing University from April 2020 to November 2023. Renal Conventional US and CEUS examination conducted less than 3 days prior to renal biopsy. The inclusion criteria were as follows: (i) LN confirmed by renal biopsy; (ii) age of more than 18 years old; (iii) body mass index (BMI) between 18.5 and 27.9. Exclusion criteria included: (i) history of contrast agent allergy; (ii) incomplete clinical data; (iii) missing or poor-quality US images; (iv) history of kidney surgery; (v) renal US parameters affected by renal cysts, hydronephrosis or masses; and (vi) pathological type classified as VI (too few cases of this type). In total, 58 patients who met the above criteria were enrolled in the study. The study design and patient flow are presented in Fig. 1.

### Clinical and laboratory data

For the included patients, demographic and clinical information such as the age, gender, BMI and the disease duration of SLE were recorded. The laboratory data collected at the time of kidney biopsy included serum creatinine (Cr), albumin levels, 24-h proteinuria, estimated glomerular filtration rate (eGFR), erythrocyte sedimentation rate (ESR) and C-reactive protein (CRP); immunologic parameters were also documented, including complement 3 (C3), complement 4 (C4), anti-double stranded DNA (anti-dsDNA) antibody, anti-nucleosome antibody (AnuA), anti-Smith antibodies (anti-Sm) and anti-SSA antibody (anti-SSA).

### Conventional US and CEUS imaging

All US examinations were performed using the same US systems (Resona 8T, Mindray, Shenzhen, China), equipped with an SC6-1 U convex transducer probe. Integral US evaluation of the kidney was performed within 3 days prior to biopsy by a single radiologist with more than 5 years of experience in abdominal ultrasonography and blinded to all the clinical and laboratory results. Patients were positioned laterally for conventional US imaging. The right kidney was scanned longitudinally and cross-sectionally using grayscale US; the kidney length, width and thickness, as well as cortical and parenchyma thickness were recorded. Color Doppler imaging was applied to measure peak systolic velocity (PSV), end-diastolic velocity (EDV) and the resistive index (RI) of the interlobar artery.

CEUS was then performed on patients in the prone position, a longitudinal section of the right kidney displaying the maximum kidney area was selected for imaging. The mechanical index was set to 0.079. A 1.0 mL dose of contrast agent (SonoVue; Bracco, Milan, Italy) was administered via bolus intravenous injection, followed by a 5 mL of normal saline flush. The transducer was manually stabilized, and patients were instructed to perform shallow breathing to minimize the variation caused by respiration. The preset settings, including gain, focus position and time-gain compensation (TGC), were kept constant throughout all examinations. Scanning continued for 3 min following the injection of contrast agent. Finally, all the CEUS images and cine loops were digitally stored for subsequent analysis.

### Imaging analysis of CEUS

**Figure 2:**
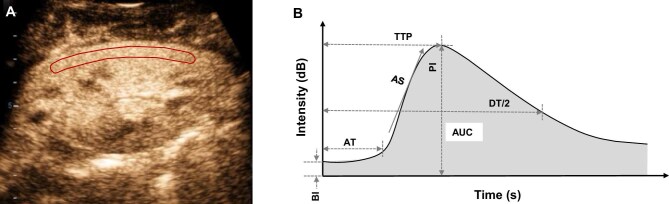
Representative image of CEUS analysis and quantitative parameters illustration. (**A**) The ROI was delineated (red line) to encompass the largest area of the renal cortex adjacent to the US probe. (**B**) Graphical illustration of a TIC with key quantitative parameters: BI, PI, AT, TTP, AS, DT/2 and AUC.

Quantitative analysis was conducted using the integrated CEUS analysis software of the Resona 8T US system. The region of interest (ROI) was manually delineated following a standardized protocol. To minimize the impact of local perfusion heterogeneities, the ROI was carefully selected to encompass the largest visible area of the renal cortex on the surface closest to the US probe (Fig. 2A). Any intermittently visualized portions of the renal cortex were excluded from the ROI to ensure measurement consistency and accuracy [18, 19].

The time-intensity curves (TICs) were automatically generated by the system, and only those achieving a goodness of fit (GOF) above 0.90 were included for further analysis. The following quantitative parameters of TICs were recorded: base intensity (BI: the baseline intensity of the renal cortex), peak intensity (PI: the maximum intensity of the curve), arrival time (AT: the time from contrast agent injection to the initial appearance of microbubbles within the ROI), time to peak (TTP: the time taken to reach the maximum intensity of the renal cortex), ascending slope (AS: the rate of intensity increase during the wash-in phase), half descending time (DT/2: the time for intensity decrease from the peak to half of its value), area under curve (AUC: the total area under the TIC curve). The characteristic parameters of a representative TIC are graphically illustrated in Fig. 2B. To account for patient-specific variations in blood volume and individual circulation, the absolute PI (∆PI = PI – BI) and absolute TTP (∆TTP = TTP – AT) were calculated.

### Repeatability of CEUS quantitative analysis

The CEUS images were retrospectively reviewed in a random order by two independent ultrasonologists, each with over 10 years of experience in renal CEUS evaluation. They independently placed the ROI on the largest visible area of the renal cortex closest to the US probe, without knowledge of any clinical or pathological information about all the patients. The TIC parameters obtained by both reviewers were subsequently assessed for reproducibility.

### Renal biopsy and histopathology

An US-guided needle biopsy was performed on the lower pole of the right kidney. The biopsy tissue was processed with hematoxylin–eosin (HE) staining and immunofluorescence staining. Two experienced renal pathologists who were blinded to all the clinical data and results of US examinations classified the renal biopsy specimens according to the ISN/RPS criteria. The renal activity (inflammation) and chronicity (fibrosis and atrophy) were scored according to the LN activity index (AI) and chronic index (CI) scoring systems developed by the National Institutes of Health. Based on the histopathological findings, Class III, IV and III/IV + V were categorized as proliferative LN, while other LN forms were classified as non-proliferative LN [20–22].

### Statistical analysis

Statistical analysis was performed using SPSS Statistics 26.0 software (IBM Corp., Armonk, NY, USA) and MedCalc Software (MedCalc, Mari-akerke, Belgium). Data are presented as mean ± standard deviation. Student’s *t*-test was used for normally distributed continuous variables, while the chi-square test and Fisher’s exact test were utilized for categorical variables. For non-parametric data, the Mann–Whitney U test or Wilcoxon signed-rank test was applied. To evaluate the reproducibility of TIC parameters obtained by different researchers, the intraclass correlation coefficient (ICC) was determined using a two-way mixed-effects model based on absolute agreement. The parameters that identified as significant differentiators in univariate analysis were included in a multivariate logistic regression analysis model to distinguish between proliferative and non-proliferative LN. The diagnostic performance of the logistic regression models was assessed through receiver operating characteristic (ROC) curves, the AUC, sensitivity, specificity, positive predictive value (PPV), negative predictive value (NPV), positive likelihood ratio (PLR) and negative likelihood ratio (NLR) with 95% confidence intervals (CIs) were calculated. A *P*-value of <.05 was considered statistically significant.

## RESULTS

### Patient characteristics

A total of 58 patients with LN confirmed by renal biopsy were included in this present study. Among them, 38 were proliferative LN, while 20 were non-proliferative LN. Table 1 summarizes the baseline characteristics and pathological results of all study participants. Our results show that there were significant statistical differences in both AI and CI between the proliferative LN group and non-proliferative LN group (*P* < .05). Whereas there was no statistical difference in age, gender, BMI and SLE disease duration between the two groups (*P* > .05).

**Table 1:** Demographic of proliferative and non-proliferative LN patients.

	All patients	Proliferative LN	Non-proliferative LN	
Variables	*n* = 58	*n* = 38	*n* = 20	*P*
Age (years)	35.66 ± 10.51	33.77 ± 9.73	39.31 ± 11.29	.087
Female gender, *n* (%)	54 (93.1)	35 (92.1)	19 (95.0)	.895
BMI (kg/m^2^)	22.57 ± 2.77	22.06 ± 2.53	24.04 ± 3.06	.081
SLE disease duration (years)	7.43 ± 5.59	6.53 ± 5.11	9.12 ± 6.19	.124
Pathologic classification, *n* (%)				
II	7 (12.1)		7 (35.0)	
III	4 (6.9)	4 (10.5)		
III + V	4 (6.9)	4 (10.5)		
IV	7 (12.1)	7 (18.4)		
IV + V	23 (39.7)	23 (60.5)		
V	13 (22.4)		13 (65.0)	
AI	4.82 ± 2.75	5.94 ± 2.49	2.08 ± 0.49	<.001^*^
CI	2.18 ± 1.37	2.47 ± 1.34	1.46 ± 1.20	.024^*^

^*^Significant value.

### Laboratory data

The laboratory values of all study participants at the time of kidney biopsy are summarized in Table 2. Our results show that there were significant statistical differences in eGFR, positive anti-dsDNA, C3 and C4 between the proliferative LN group and non-proliferative LN group (*P* < .05). Whereas there was no statistical difference in 24-h proteinuria, serum albumin, Cr, ESR, positive anti-Sm, positive anti-SSA, positive AnuA and CRP between the two groups (*P* > .05).

**Table 2:** Laboratory values at the time of kidney biopsy.

	All patients	Proliferative LN	Non-proliferative LN	
Variables	*n* = 58	*n* = 38	*n* = 20	*P*
24 h proteinuria (g)	4.21 ± 3.55	4.09 ± 3.26	4.50 ± 4.29	.959
Serum albumin (g/L)	30.50 ± 6.24	30.30 ± 5.88	30.98 ± 7.29	.742
Serum Cr (mmol/L)	68.22 ± 27.77	73.13 ± 28.52	56.16 ± 22.49	.063
eGFR (mL/min/1.73 m^2^)	109.04 ± 39.07	100.05 ± 33.92	131.15 ± 43.36	.014^*^
ESR (mm/h)	41.24 ± 31.09	38.44 ± 32.79	48.15 ± 26.34	.348
Anti-dsDNA positive (%)	38 (65.5)	30 (78.9)	8 (40.0)	.003^*^
Anti-Sm positive (%)	28 (48.3)	16 (42.1)	12 (60.0)	.195
Anti-SSA positive (%)	39 (67.2)	25 (65.8)	14 (70.0)	.745
AnuA positive (%)	37 (63.8)	26 (68.4)	11 (55.0)	.312
C3 (g/L)	0.74 ± 0.31	0.66 ± 0.24	0.96 ± 0.39	.025^*^
C4 (g/L)	0.14 ± 0.11	0.11 ± 0.08	0.22 ± 0.14	.032^*^
CRP (mg/L)	4.97 ± 5.83	4.63 ± 5.59	5.78 ± 6.55	.269

^*^Significant value.

### Repeatability of CEUS parameters

To evaluate the reproducibility of TIC parameters obtained by different researchers, interobserver agreement was evaluated. It was found that there was good interobserver agreement of ∆PI, ∆TTP, AS, DT/2 and AUC between different researchers (Table 3).

**Table 3:** Repeatability of CEUS parameters.

Parameter	ICC	95% CI
∆PI	0.944	0.897–0.970
∆TTP	0.940	0.890–0.967
AS	0.873	0.775–0.930
DT/2	0.913	0.843–0.953
AUC	0.987	0.976–0.993

∆PI = PI – BI; ∆TTP = TTP – AT.

### Conventional US and CEUS parameters

**Figure 3:**
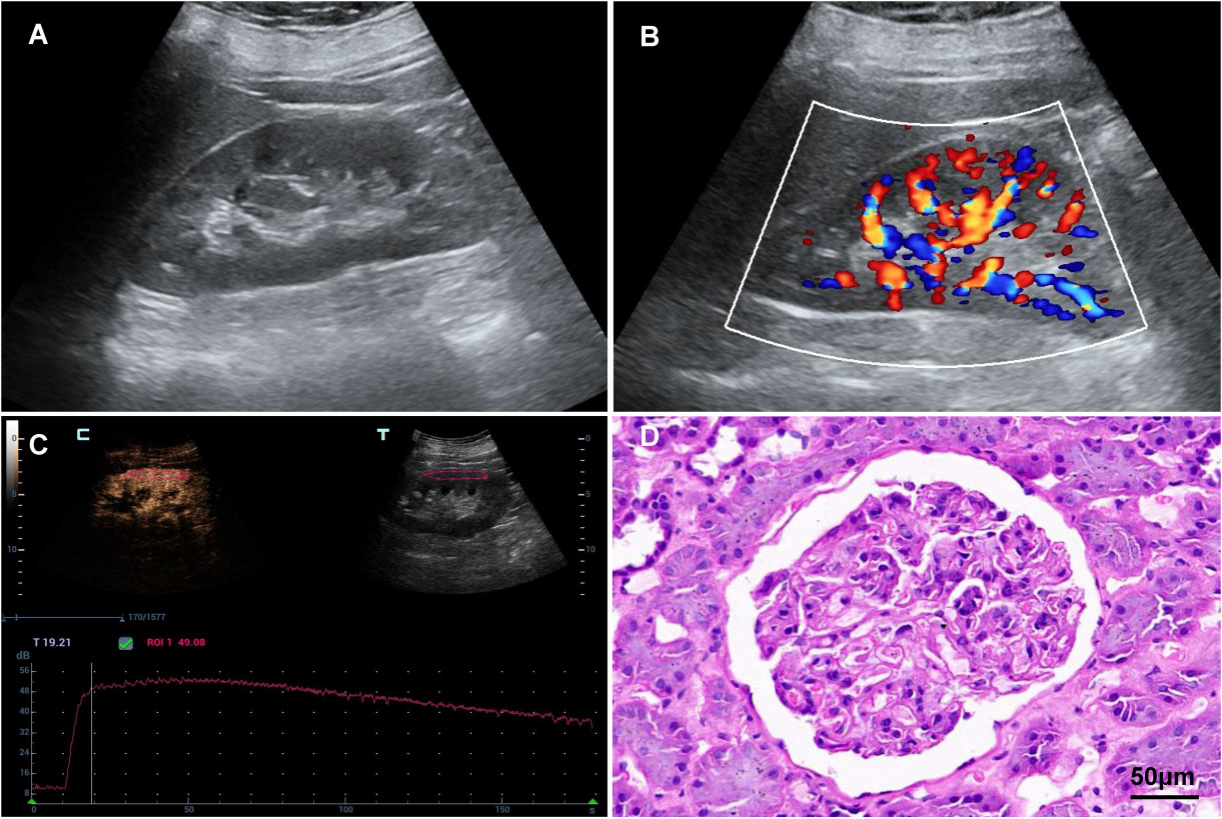
Imaging and histopathological findings of a 37-year-old woman with non-proliferative LN. (**A**) Grayscale US imaging of the right kidney. (**B**) Color Doppler imaging of the right kidney. (**C**) TIC of ROI in the right kidney cortex obtained from CEUS. (**D**) The histopathologic result indicating ISN/RPS Class II.

**Figure 4:**
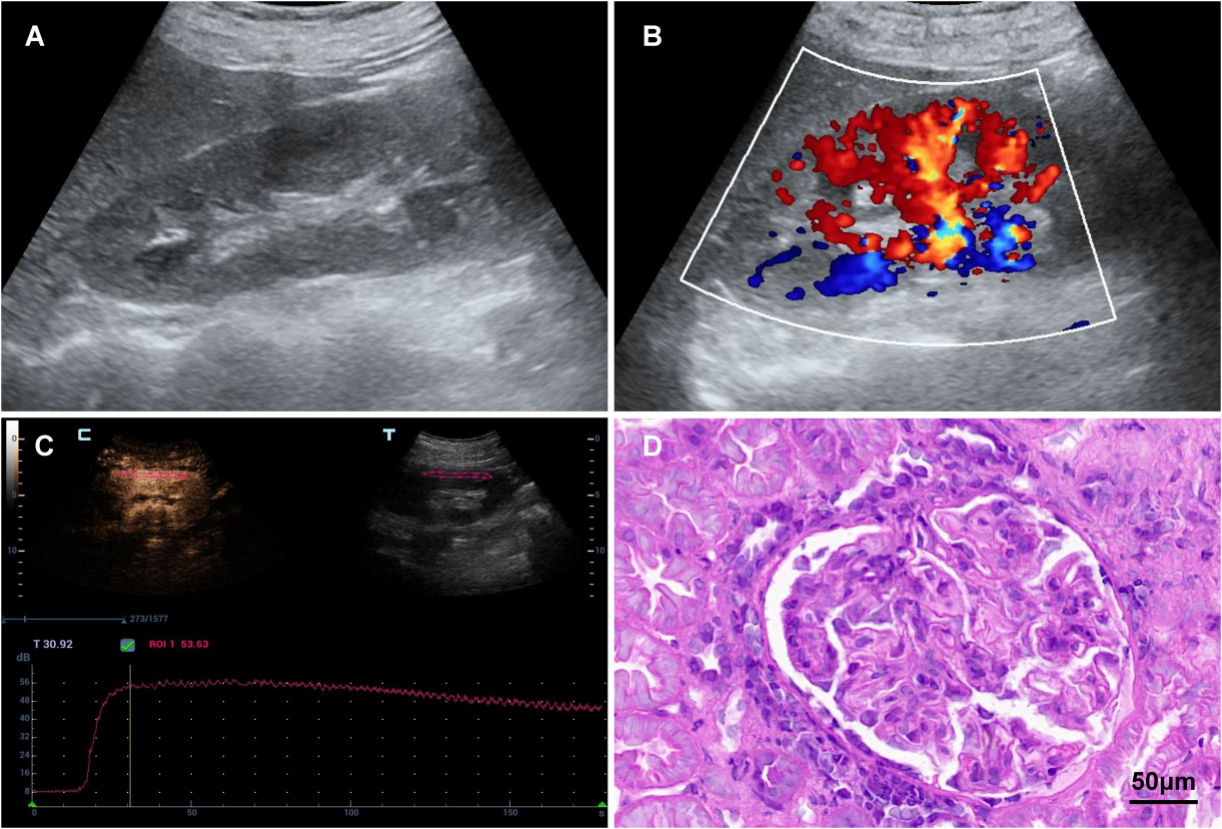
Imaging and histopathological findings of a 39-year-old woman with proliferative LN. (**A**) Grayscale US imaging of the right kidney. (**B**) Color Doppler imaging of the right kidney. (**C**) TIC of ROI in the right kidney cortex obtained from CEUS. (**D**) The histopathologic result indicating ISN/RPS Class IV + V.

Analysis of conventional US parameters revealed no significant differences between the proliferative and non-proliferative LN groups in terms of renal size, cortical thickness, parenchymal thickness, PSV, EDV and RI (*P* > .05). However, CEUS parameters showed that the ΔTTP, DT/2 and TIC-AUC values were significantly higher in the proliferative LN group compared with the non-proliferative LN group (*P* < .05). There was no significant difference between the two groups in ΔPI, AT and AS values (*P* > .05) (Figs 3 and 4, Table 4).

**Table 4:** Conventional US and CEUS parameters of non-proliferative and proliferative LN patients.

	All patients	Proliferative LN	Non-proliferative LN	
Parameter	*n* = 58	*n* = 38	*n* = 20	*P*
Conventional US				
Renal length (mm)	111.20 ± 7.40	110.31 ± 6.79	113.38 ± 8.64	.211
Renal width (mm)	47.89 ± 5.17	46.97 ± 4.43	50.15 ± 6.27	.060
Renal thickness (mm)	62.44 ± 5.71	62.16 ± 5.87	63.15 ± 5.46	.601
Cortical thickness (mm)	7.97 ± 1.44	7.75 ± 1.44	8.58 ± 1.31	.087
Parenchyma thickness (mm)	16.00 ± 1.82	15.81 ± 1.79	16.50 ± 1.88	.269
PSV (cm/s)	35.26 ± 8.49	35.06 ± 9.48	35.93 ± 4.28	.818
EDV (cm/s)	15.75 ± 4.12	15.61 ± 4.47	16.20 ± 2.87	.748
RI	0.56 ± 0.04	0.57 ± 0.04	0.55 ± 0.04	.424
CEUS				
∆PI (dB)	32.62 ± 4.71	33.44 ± 3.91	30.61 ± 5.96	.067
AT (s)	0.26 ± 0.05	0.27 ± 0.05	0.26 ± 0.05	.469
∆TTP (s)	46.05 ± 8.06	47.98 ± 6.35	41.31 ± 10.00	.041^*^
AS	0.659 ± 0.10	0.645 ± 0.08	0.692 ± 0.12	.136
DT/2 (s)	163.90 ± 21.24	169.41 ± 14.97	150.34 ± 28.19	.035^*^
AUC	8032.96 ± 924.70	8306.30 ± 743.67	7237.79 ± 970.72	.006^*^

^*^Significant value.

∆PI = PI – BI; ∆TTP = TTP – AT.

### Logistic regression analysis and ROC curve

**Figure 5:**
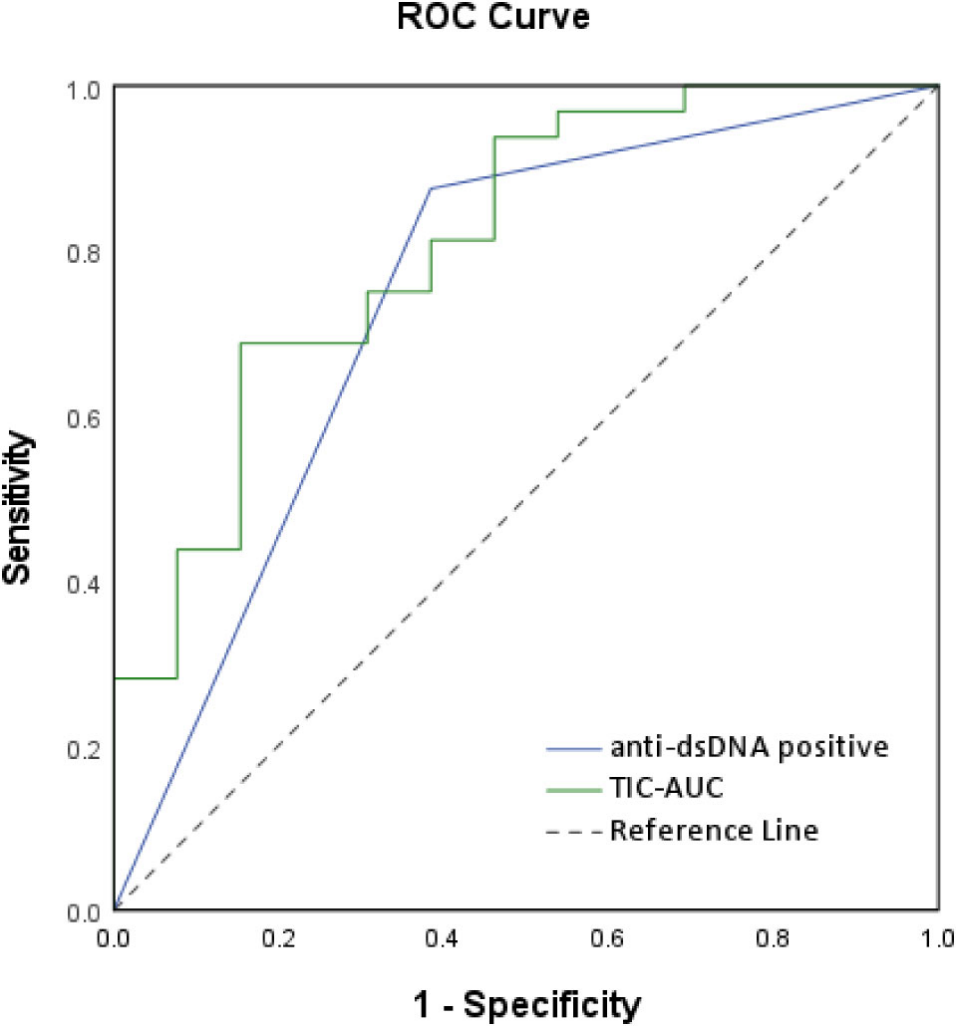
ROC curves of the logistic regression models for predicting proliferative LN.

In the multivariate logistic regression analysis incorporating clinical and CEUS quantitative parameters in the model, we found the anti-dsDNA positive and AUC in TIC were the significant predictors of proliferative LN (*P *< .05). The significant predictors and their corresponding odds ratios are presented in Table 5. ROC curve analysis (Fig. 5) was performed to evaluate the diagnostic performance of these predictors. For anti-dsDNA positive, the AUC was 0.745, with a sensitivity of 87.5% and specificity of 61.5% in predicting proliferative LN. Using a TIC-AUC cutoff value of 8049.0, the AUC was 0.810, sensitivity was 68.8% and specificity was 84.6% for predicting proliferative LN. Detailed diagnostic performance metrics, including PPV, NPV, PLR and NLR, along with their 95% CIs, are summarized in Table 6.

**Table 5:** Multivariate logistic regression analysis for the prediction of proliferative LN.

						95% CI for Exp(B)	
	B	SE	Wald	*P*-value	Exp(B)/OR	Lower	Upper
TIC-AUC	1.813	0.729	6.179	.013	6.129	1.467	25.601
Anti-dsDNA positive	2.923	0.989	8.732	.003	18.596	2.676	129.245

SE, standard error; OR, odds ratio.

**Table 6:** Diagnostic performance of the variables for predicting proliferative LN.

	AUC	Sensitivity	Specificity	PPV	NPV	PLR	NLR
TIC-AUC	0.810 (0.668–0.952)	68.8% (50.0%–83.9%)	84.6% (54.6%–98.1%)	91.7% (75.1%–97.6%)	52.4% (38.5%–65.9%)	4.47 (1.22–16.33)	0.37 (0.21–0.65)
Anti-dsDNA positive	0.745 (0.570–0.920)	87.5% (71.0%–96.5%)	61.5% (31.6%–86.1%)	84.8% (73.6%–91.9%)	66.7% (42.1%–84.6%)	2.28 (1.13–4.58)	0.20 (0.074–0.56)

## DISCUSSION

In this study, we analyzed the differences between proliferative and non-proliferative LN patients concerning clinical characteristics, laboratory data and US parameters of kidneys. Notably, we investigated the potential of CEUS quantitative parameters in predicting the pathological classification of LN, comparing their effectiveness to conventional clinical and laboratory markers. Our findings indicate that CEUS parameters, particularly the AUC of the TICs, provide valuable information into the distinguishing between proliferative and non-proliferative LN. This demonstrated the utility of CEUS as a non-invasive, convenience and effective tool for predicting LN pathology.

Our sample was similar to other cohorts with female predominance and age distribution [23]. Additionally, the most common LN histological classes in our study were proliferative LN (Class III, IV and III/IV + V), this distribution is similar to the findings from other kidney biopsy–based LN studies. In many centers for SLE treatment, kidney biopsy is recommended for patients presenting with higher levels of proteinuria, which increasing the probability of detecting proliferative LN in biopsy samples [24, 25].

In our study, the proliferative LN group exhibited significantly lower levels of eGFR, C3 and C4, and a higher likelihood of positive anti-dsDNA. In the multivariate analysis, anti-dsDNA positive was associated with a higher probability of the patient being classified with proliferative LN compared with non-proliferative LN. This finding is consistent with previous studies and may be attributed to heightened immune system activation and increased immune complexes formation in patients with proliferative forms of LN [21]. We also evaluated the diagnostic performance of other conventional laboratory markers, including 24-h proteinuria, C3 and C4, previously linked to proliferative LN [26]. Comprehensive diagnostic metrics, including AUC, sensitivity, specificity, PPV, NPV, PLR and NLR, with the associated ROC curves provided in the Supplementary Materials. The AUC values for 24-h proteinuria, C3 and C4 were 0.507, 0.719 and 0.707, respectively. The limited diagnostic value of 24-h proteinuria may be due to the fact that kidney biopsies in these cases were likely performed because of elevated proteinuria levels and clinical suspicion of a proliferative component, which could introduce a selection bias. While low C3 and C4 demonstrated moderate diagnostic accuracy, their lack of significance in multivariate analysis suggests that these biomarkers may have limited independent predictive value for proliferative LN when adjusted for other covariates.

The results showed that conventional US parameters, including kidney size and RI, failed to show significant differences between proliferative and non-proliferative LN groups, while CEUS-derived TIC parameters, including ΔTTP, DT/2 and AUC, were able to distinguish between the two groups. Notably, the AUC of TIC emerged as a strong predictor for proliferative LN. The difference in renal blood perfusion observed through CEUS may reflect the more intense inflammatory and immune-mediated processes occurring in proliferative LN, which are not captured by conventional US. Previous studies have confirmed the value of CEUS in assessing microvascular perfusion in various kidney diseases, including acute kidney injury and chronic kidney disease. Our findings further extend the application of CEUS to LN, especially in differentiating between the proliferative and non-proliferative forms of the disease.

Renal vascular lesions are common pathological manifestations in LN [27]. When these lesions occur, immune complexes deposit on the walls of renal blood vessels, leading to luminal stenosis or occlusion [28]. This increases the resistance of afferent arterioles, slowing blood perfusion and resulting in delayed TTP. The AUC of TIC reflects the amount of perfusion and clearance within the region of interest over a unit of time, which is influenced by factors such as distribution volume, blood flow velocity and the perfusion time of the microbubble contrast agent [29]. In some studies regarding renal cortical perfusion, the AUC of TIC was considered more sensitive than other parameters [30]. In our study, the prolongation of DT/2 and the increase in TIC-AUC may be associated with the increased vascular endothelial growth factor expression during the inflammatory phase of LN, which promotes the proliferation of the surrounding capillary network and increases the effective vascular bed area [31, 32]. At the same time, the formation of large amounts of immune complexes and vascular wall damage alters the hemodynamic characteristics of blood flow within the vessel lumen, leading to blood stasis and relatively prolonged perfusion time. Compared with non-proliferative LN, proliferative LN is associated with higher disease activity and more severe intracapillary circulation impairment. Therefore, we hypothesize that the quantitative parameters obtained through CEUS in LN can reflect the pathological classification.

Previous studies on CEUS-based renal cortical perfusion quantification have used varying sizes and numbers of ROI, including the use of three small (5 × 5 mm^2^) ROIs in some studies [33, 34]. In our study, we implemented a standardized protocol for all ROI placements to minimize variability. Specifically, we found that selecting a larger area of ROI covering the visualized renal cortex effectively reduced regional heterogeneity in renal perfusion and produced smoother TICs. This strategy could potentially mitigate the impact of lateral shift variation. Furthermore, we observed good interobserver agreement in the CEUS perfusion parameters between different researchers, which reflects reduced heterogeneity and reinforces the validity of our analytical approach.

Although renal biopsy remains the gold standard for diagnosing LN, its invasive nature and potential risks, such as bleeding, particularly in SLE patients with coagulation abnormalities, make non-invasive imaging techniques an attractive alternative for disease evaluation. Among these, CEUS stands out for its non-invasive nature, high repeatability and real-time functional assessment capabilities. CEUS not only facilitates the ongoing assessment of renal pathology in LN patients, but also offers a dynamic and comprehensive view of renal involvement compared with static biopsy samples. In our study, the combination of CEUS parameters with clinical and laboratory markers, such as anti-dsDNA antibody, in multivariate logistic regression analysis significantly enhanced the diagnostic performance for predicting proliferative LN. This was further supported by the ROC curve analysis, which demonstrated that TIC-AUC and anti-dsDNA positive exhibited good sensitivity and specificity, suggesting their complementary roles in identifying patients with proliferative LN.

Our study has several limitations. First, the sample size was relatively small, particularly for non-proliferative LN subgroup (*n* = 20), in which only 7 Class II cases were included. It should be noted that our grouping method used in this study does not negate the important clinical distinctions between these pathological classes. Future studies with larger subclass-specific cohorts (Class II vs V vs III/IV) are needed to further validate the predictive value of CEUS parameters in LN classification and to provide more targeted guidance for clinical practice. Additionally, our study was retrospective in nature, and prospective studies will be essential to confirm the potential of CEUS in predicting long-term renal outcomes in LN patients. Furthermore, since our study only focused on one specific CEUS system and protocol, the reproducibility of these findings using different US systems and in other clinical settings should be explored in future multicenter research.

Another limitation is the dependence on the ISN/RPS classification system, which does not fully account for vascular lesions such as thrombotic microangiopathy (TMA). We did not specifically analyze TMA or other vascular lesions in this study; future studies should incorporate detailed vascular lesion assessments to better understand their impact on CEUS parameters and further validate the utility of CEUS in LN evaluation.

In conclusion, our study preliminary demonstrates the value of CEUS quantitative parameters, particularly TIC-AUC, effectively complement traditional laboratory markers in predicting proliferative LN. As a non-invasive, convenient and effective approach, CEUS could provide additional diagnostic information, particularly in LN patients with contraindications to biopsy or those requiring frequent monitoring. Further validation in larger and more diverse patient cohorts is needed to determine its potential to reduce reliance on invasive biopsies in clinical practice.

## Supplementary Material

sfaf314_Supplemental_File

## Data Availability

Data are available upon reasonable request.
