# Supplementary material for: Diagnostic utility of contrast-enhanced ultrasound parameters in classifying lupus nephritis
Source: Clin Kidney J. 2025 Oct 8;18(11):sfaf314. doi: 10.1093/ckj/sfaf314 (PMC12605818; doi:10.1093/ckj/sfaf314)
Supplement: sfaf314_Supplemental_File [file sfaf314_supplemental_file.pdf]

Table. Diagnostic performance of the variables for predicting proliferative LN.

	AUC	Sensitivity	Specificity	PPV	NPV	PLR	NLR
TIC-AUC	0.810(0.668~0.952)	68.8%(50.0%~83.9%)	84.6%(54.6%~98.1%)	91.7%(75.1%~97.6%)	52.4%(38.5%~65.9%)	4.47(1.22~16.33)	0.37(0.21~0.65)
anti-dsDNA positive	0.745(0.570~0.920)	87.5%(71.0%~96.5%)	61.5%(31.6%~86.1%)	84.8%(73.6%~91.9%)	66.7%(42.1%~84.6%)	2.28(1.13~4.58)	0.20(0.074~0.56)
C3	0.719(0.565~0.842)	90.6%(75.0%~98.0%)	53.9%(25.1%~80.8%)	82.9%(72.7%~89.8%)	70.0%(41.5~88.5%)	1.96(1.08~3.57)	0.17(0.053~0.57)
C4	0.707(0.552~0.833)	81.3%(63.6%~92.8%)	69.2%(38.6%~90.9%)	86.7%(73.9%~93.7%)	60.0%(40.1%~77.1%)	2.64(1.15~6.07)	0.27(0.12~0.61)
24h proteinuria	0.507(0.354~0.659)	59.4%(40.6%~76.3%)	53.9%(25.1%~80.8%)	76.0%(62.2%~85.9%)	35.0%(21.9%~50.9%)	1.29(0.67~2.47)	0.75(0.39~1.45)

LN: lupus nephritis; TIC: time-intensity curves; AUC: area under curve; anti-dsDNA: anti-double stranded deoxyribonucleic antibody; C3: complement 3; C4: complement 4; PPV: positive predictive value; NPV: negative predictive value; PLR: positive likelihood ratio; NLR: negative likelihood ratio

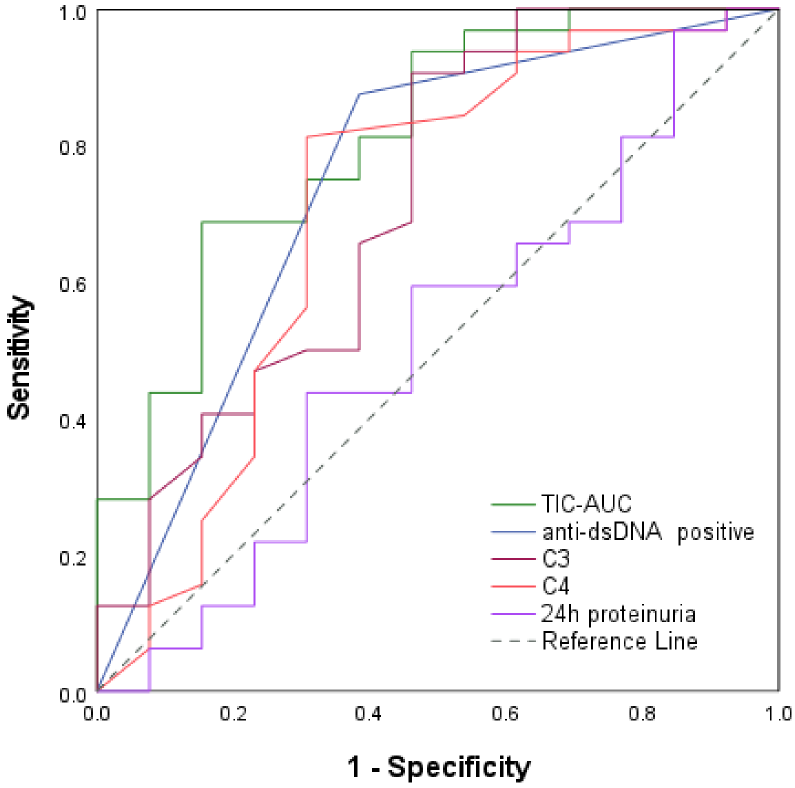

Figure. Receiver operating characteristic (ROC) curves of the logistic regression models for predicting proliferative lupus nephritis (LN).
